# Sex ratio effects on reproductive strategies in humans

**DOI:** 10.1098/rsos.140402

**Published:** 2015-01-14

**Authors:** Ryan Schacht, Monique Borgerhoff Mulder

**Affiliations:** 1 Department of Anthropology, University of Utah, Salt Lake City, UT 84112, USA; 2 Department of Anthropology, University of California-Davis, Davis, CA 95616, USA; 3 Graduate Group in Ecology, University of California-Davis, Davis, CA 95616, USA; 4 Center for Population Biology, University of California-Davis, Davis, CA 95616, USA

**Keywords:** sex ratio, competition, sexual selection, sex roles, Makushi–Guyana

## Abstract

Characterizations of coy females and ardent males are rooted in models of sexual selection that are increasingly outdated. Evolutionary feedbacks can strongly influence the sex roles and subsequent patterns of sex differentiated investment in mating effort, with a key component being the adult sex ratio (ASR). Using data from eight Makushi communities of southern Guyana, characterized by varying ASRs contingent on migration, we show that even within a single ethnic group, male mating effort varies in predictable ways with the ASR. At male-biased sex ratios, men's and women's investment in mating effort are indistinguishable; only when men are in the minority are they more inclined towards short-term, low investment relationships than women. Our results support the behavioural ecological tenet that reproductive strategies are predictable and contingent on varying situational factors.

## 2. Introduction

Recent advances in the evolutionary analysis of reproductive strategies suggest that the adult sex ratio (ASR) [1–3], together with certainty of parentage [4,5] and intrasexual variability in quality [6] are key to generating predictions of sex-differentiated mating and reproductive behaviour. Here, we explore how these current formulations of sexual selection theory can shed light on the diversity of human sex roles. More specifically, we use natural variation in ASR among different communities in an indigenous Amerindian population in Guyana to explore how one particular aspect of human reproductive effort, investment in mating (a key aspect of reproductive strategy insofar as effort invested in mating trades off directly with parental effort; [7,8], but see [9]), reflects an environmental gradient in the relative abundance of males and females. Darwin made a famous distinction between men's and women's mating strategies, between choosy, coy females and ardent, promiscuous males [10]. While Darwin was careful to nuance this viewpoint, Bateman's support of this distinction with his fruit fly experiments became central to sexual selection theory ([11], but see [12–14]) and to the attribution of sex roles to sex differences in parental investment (PI) [15]. This distinction in sex roles strongly shaped the study of animal breeding systems (e.g. [16]) as well as influential work in the evolutionary social sciences (e.g. [17–19]). Yet, as also anticipated by both Darwin [20] and Trivers [15], empirical studies of both non-humans and humans reveal extraordinary flexibility in mating and investment behaviour, both within and between the sexes. Reproductive strategies are clearly not an invariant, species-specific characteristic, but rather facultative responses to individual- and population-level social and ecological circumstances (e.g. [21–23]), rendering conditional mating strategies optimal [19,24].

Social scientists should welcome this attention to variable sex roles in so far as cross-cultural variability in human reproductive and mating behaviour seems to be the rule rather than exception [25]. For example, across the ethnographic record, human societies can range from polyandrous to polygynous mating patterns [26], same-sex marriage can be institutionalized as with woman-to-woman unions in East Africa [27] and men can spend considerable amounts of time and effort in beautifying themselves as in West Africa [28]. As in non-humans [29], patterns of sexual selection on men and women can be highly variable [25,30,31].

Until recently, the study of sex-differentiated reproductive behaviour has relied on the long-standing model of sexual selection developed by Trivers [15]. This model links sex roles directly to the differential investment in young by males and females. In its simplest form, this model posits that because males invest less initially (in sperm), they have a higher potential reproductive rate (PRR) [32] and benefit more from mating multiply than do females. As a consequence, selection typically would favour mate-seeking and competitive behaviour in males, and heavy investment in parental care in females. In an extension of this model, Emlen & Oring [33] claim that as the number of available males to available females rises (an increasing operational sex ratio, OSR), so does competition among males over the few available females. Predictions based on OSR have been used in many studies of human mating systems to predict more violent and competitive male behaviour when males are in abundance (reviewed in [34]).

To explore variability in sex roles, we turn to a newer approach within sexual selection theory in which patterns of care and competition coevolve, through evolutionary feedbacks, with the sex-specific intensity of sexual selection [35]. A key feature of this approach is the game theoretical approach it takes [2] to modelling the sex-structured pay-offs to reproductive behaviour according to the relative scarcity of the sexes (e.g. [3]). While certainty of parenthood [5] and variability in quality [6] play a role in structuring sex differences in reproductive effort, a key claim of the newer approach is that that for males, under most conditions, the profitability of investment in mating effort increases when there are more, not fewer, mating opportunities available. Those males that pursue a mating-effort intensive strategy when mates are rare may find themselves spending longer periods in between reproductive events than if they were to stay with their initial partner and provide parental care [2] or mate guard [3]. Thus, when men are in abundance and surrounded by competitors, they should reduce, not increase, mating effort [36]. In species with female choice of mates, they should alter their behaviour to match that which is desired by females and therefore offer more, not less, PI. Note that this is a prediction more in line with mating market models which take a frequency-dependent approach to sex roles [37–39]. Optimal behaviour depends on the dynamics of evolutionary feedbacks between scarcity, sex-structured pay-offs to mating strategies, time spent in providing parental care and the mortality associated with parental care and mating competition.

In the behavioural ecology of other species, both empirical work [40–42] and modelling [43–46] have demonstrated the importance of scarcity [39] of one sex in relation to the other. For example, in soapberry bugs ( *Jadera haematoloma*), Carroll & Corneli [47] find that male behaviour responds facultatively to the availability of females; when males are in abundance and females in demand, males practice mate guarding as a means to ensure paternity certainty rather than seeking multiple mates through intrasexual competition. Similarly, Liker *et al.* [41] recently showed that in shorebirds with male-biased ASRs, female–female competition and male paternal care (and even polyandry) are present, as in the jacanas (Jacanidae) and greater painted-snipe (*Rostratula benghalensis*); whereas species with polygyny, such as the ruff (*Philomachus pugnax*), have female-biased ASRs. More generally, across animal taxa, monogamy is associated with male-biased sex ratios and low variance in male reproductive success, whereas polygyny is associated with female-biased sex ratios and high variance in male reproductive success [2].

Here, we explore how investment in mating effort differs between the sexes in a human population. We test two models. The first (model 1) tests the hypothesis that men should consistently invest more in mating effort than women owing to their higher PRR and greater benefits they receive from pursuing multiple mates (H1). Following Trivers [15], it is argued that higher mating effort in men than women ‘should be consistently observed across cultures, in part because of the fundamental differences in evolved reproductive strategies of men and women’ [48], p. 249. Furthermore, according to this line of thinking (following [33]), males should invest more in mating effort when they outnumber females in the mating pool. We use the second model (model 2), which includes terms for sex, ASR and their interaction, to test the more specific prediction that the differences between the sexes in their reproductive strategies are conditioned on the availability of partners, or market principles of supply and demand; males and females are both expected to invest more in mating effort when scarce because members of the rarer sex have more potential partners to mate with [49].

**Figure 1.**
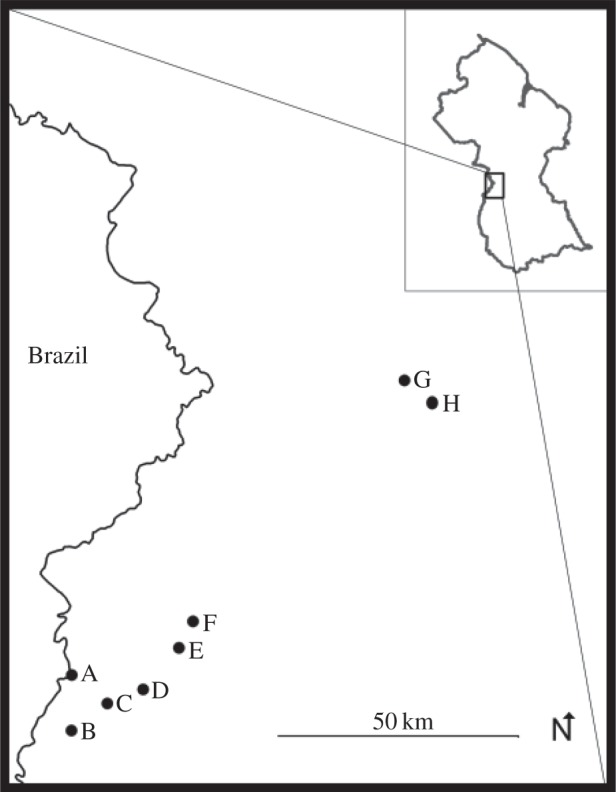
Map of Guyana and field site locations.

To test these models, we capitalize on natural variation in ASR within a single ethnic group, the Makushi of Guyana living in the Rupununi savannah (figure 1), where the relative numbers of men and women in a village (table 1) reflect subsistence and employment conditions that are quite stable over time. The principle drivers of outmigration for men are mining, cattle ranching, agricultural work and logging, activities which occur mainly in more remote areas of the Rupununi or in the forested regions at the centre of the country, whereas women are attracted to urban areas (such as the capital of Roraima in neighbouring Brazil) and the larger interior Guyanese towns (such as Lethem) in search of shop and domestic work [50]. Marriage among the Makushi is endogamous, with mates usually selected from within the village community. Thus, community ASR strongly structures marital options. Marriage is also monogamous. Prior to marriage, a man must perform bride service, typically garden labour and fishing, for the household of his prospective bride's parents. After marriage, residence is matrilocal in close proximity to the bride's family [51].

**Table 1.** Descriptive statistics of each community.

community	ASR	men : women (18–45)	no. men interviewed	no. women interviewed	community population
A	0.93	125 : 135	29	29	745
H	1.11	70 : 63	15	15	415
F	1.13	70 : 62	15	15	407
E	1.16	87 : 75	18	19	596
G	1.22	73 : 60	20	20	432
D	1.33	80 : 60	19	19	406
B	1.35	27 : 20	15	15	162
C	1.43	57 : 40	18	20	310

We focus on investment in mating effort, which we measure using the sociosexual orientation inventory (SOI) [52,53]. These sociosexuality scores are used to evaluate an individual's willingness to engage in uncommitted sexuality activity. Individuals who score high on the SOI scale (termed ‘less restricted’) evince a reproductive strategy of multiple short-term relationships and low PI; those scoring low on the SOI scale (‘more restricted’) prefer longer term relationships with higher PI [54–56]. The SOI is a tool that has been shown in previous work (e.g. [53,57,58]) to be a reliable indicator of individual allocation of effort to mating. We explore how an individual's sex (model 1), and the ASR of his or her community (model 2), contributes to an understanding of the reproductive strategies of men and women among the Makushi as measured by the SOI. In so doing, we make, to our knowledge, the first explicit evaluation of the theoretical perspective for gender variability in reproductive strategies among humans.

## 3. Results

**Figure 2.**
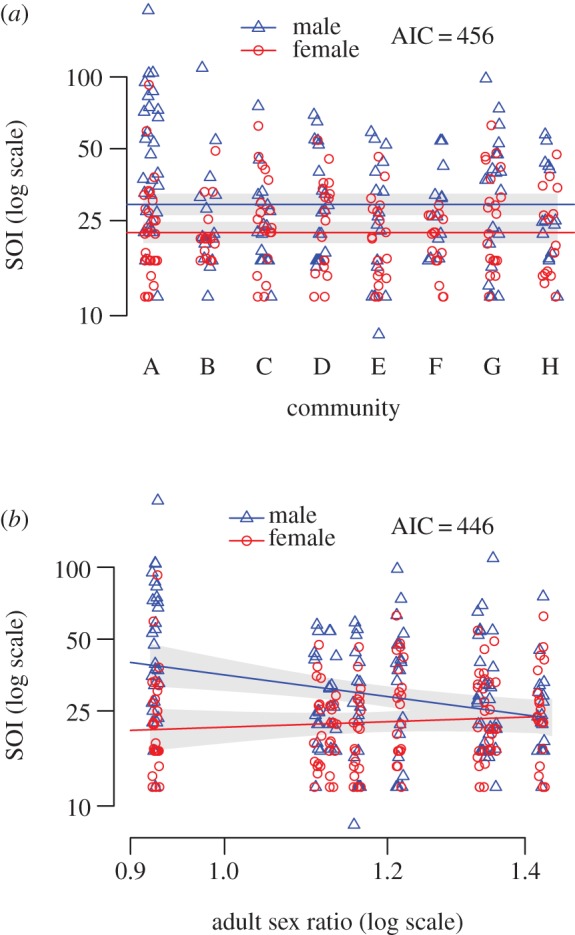
Sociosexual orientation inventory (SOI) scores by (*a*) sex and community (model 1) and by (*b*) sex and community ASR (model 2).

Our sample is composed of 300 individuals from eight rural communities (see Material and methods for sampling frame and strategy) in the Rupununi region of Guyana. These communities exhibited a wide range in ASR values (0.9–1.4; table 1), resulting from spatially heterogeneous employment opportunities for men and women. We first test the hypothesis that men have a less restricted sociosexuality than women (model 1). Our statistical model includes a fixed effect for sex and a random effect for community and is constructed to assess the evidence for consistent sex differences in reproductive strategy as measured by the individual's sociosexual score. The fixed effect measures a stable contrast between males and females and the random effect allows for community-to-community heterogeneity in sociosexuality scores, as dictated by the multilevel structure of the dataset (see Material and methods). Analysis of this model finds that men generally have higher sociosexuality scores (are less restricted) across communities, indicating that in general Makushi men show a greater willingness to engage in uncommitted sex than do women. This provides initial support for model 1 (figure 2*a*).

Our second model incorporates community-level ASR (model 2). It entails the expectation that when one sex is in oversupply, variations in male and female reproductive strategies will respond as expected by market principles of supply and demand. Specifically, we predict that when men are in short supply (female-biased ASR), they can be more demanding, that is they will have a less restricted sociosexuality than when they are in oversupply (male-biased ASR). Conversely, when women are in oversupply (female-biased ASR), they will be more restricted in their sociosexuality than women at a male-biased ASR. The statistical model includes as a predictor, the community-level ASR, as well as the interaction of ASR and sex. We include this interaction to allow the relationship between sociosexuality scores and ASR to be different for men and women.

Analysis of this model shows diversity in sociosexuality scores across ASR. When there are fewer men (female-biased ASR), men's sociosexuality scores are not only higher than women's, but are also higher than those of men living in communities where men are relatively abundant (figure 2*b*), meaning that at female-biased sex ratios men show greater tendencies towards promiscuity. Men show higher sociosexuality scores when they are in the minority, as predicted by model 2. For women, we do not detect an effect of ASR on sociosexuality (table 2). In short, men appear to increase their willingness to engage in uncommitted sex in response to a surfeit of women and to become less keen to engage in uncommitted sex when men are abundant; women by contrast appear indifferent to changes in the ASR. It is notable that at a male-biased ASR, male and female sociosexuality scores are indistinguishable.

**Table 2.** Parameter estimates and summaries for the effects of sex (model 1) and of sex, ASR and the ASR×sex interaction (model 2) on SOI scores (standard errors in parentheses), including community-level effects.

	model 1	model 2
intercept	3.10(0.05)	3.06(0.08)
sex=male	0.27(0.06)	0.50(0.09)
logASR		0.29(0.36)
logASR×sex=male		−1.43(0.40)
AIC	456	446
var(community)	0.09	0.08
var(residual)	0.50	0.49

Adding ASR (model 2) greatly improves our understanding of the diversity in sociosexuality scores across the eight communities over the model which only includes sex (model 1). The Akaike information criterion (AIC) model score is lowered by 10 (table 2). To counter the possibility that the ASR effect is confounded by relationship status or age, we added each (separately) as a main effect to both models (model 1+ and model 2+ include the term relationship status and model 1++ and model 2++ include age). As might be expected, single individuals have higher sociosexuality scores than those that are partnered; nevertheless, the association of sociosexuality with community ASR and sex remains, and the relative evidence for model 2+ is much stronger than for model 1+ (lowered by an AIC difference of 13; table 3). Adding age to the models, however, has little effect on the goodness of fit for either model (table 3).

**Table 3.** Parameter estimates and summaries for the effects of sex and relationship status (model 1+), sex, ASR, the ASR×sex interaction and relationship status (model 2+), sex and age (model 1++), and of sex, ASR, the ASR×sex interaction and age (model 2++) on SOI scores (standard errors in parentheses), including community-level effects.

	model 1+	model 2+	model 1++	model 2++
intercept	3.06(0.05)	3.00(0.08)	3.45(0.12)	3.39(0.13)
sex=male	0.25(0.06)	0.49(0.08)	0.29(0.06)	0.51(0.09)
logASR		0.42(0.36)		0.30(0.36)
logASR×sex=male		−1.52(0.39)		−1.39(0.39)
relationship status	0.30(0.07)	0.31(0.07)		
age			−0.01(0.00)	−0.01(0.00)
AIC	445	433	457	448
var(community)	0.09	0.09	0.09	0.09
var(residual)	0.49	0.48	0.49	0.48

## 4. Discussion

Our findings show, among the Makushi, the mating and investment behaviour of men varies in predictable ways with the ASR. While a larger sample of villages would of course add strength to our inference, we demonstrate that at male-biased sex ratios, men's and women's willingness to engage in uncommitted sex are indistinguishable and that when men are in the minority they are more inclined towards indiscriminate mating than when in the majority. This is not to conclude that sex differences are not important in Makushi reproductive strategies, after all a term for sex is maintained in the best fit model, but to highlight the fact that the reproductive strategies of men are strongly affected by the ASR. Here, we discuss: (i) the implications of this approach for the evolutionary study of gender, (ii) the relationship of our work with previous work, (iii) the importance of studying within-population variation, (iv) the issue of time scales, and (v) outstanding questions.

First, with respect to implications for the evolutionary study of gender, our findings reject simplistic labelling of reproductive roles by gender based on inherent sex differences in PI. In this respect, contemporary evolutionary anthropology is advanced well beyond overly simplistic interpretations of the work of Darwin and Bateman [59]. Our results challenge sexual stereotyping that can arise from models inappropriately linking men's and women's reproductive strategies to the constraints of parental care [10,60,61]. We advance these concerns by bringing a rigorous theoretical model predicated on coevolutionary dynamics to the study of reproductive strategy.

Second, previous evolutionary analyses of human sexual strategies have acknowledged the existence of variability in reproductive strategies [19,48,62]. In some cases, they have attributed inter- or intra-population variability in mating effort to exogenous variables such as pathogen prevalence [63]. For example, in a study of men's and women's preferences when exposed to indicators of pathogens, both genders were more concerned with attractiveness and symmetry in high disease rather than low disease conditions. They appear to use attractiveness as an indicator of underlying health and immune functioning [64]. In other cases, investigators attribute intrasexual variation in mating strategies to ‘strategic pluralism’ [19], providing often innovative arguments about how multiple factors, including relationship length, individual quality variation and social context affect the optimal reproductive strategies for men and women.

Where such evolutionary studies have considered sex ratio, they nevertheless assume that sex ratios will impact mating strategies as proposed in older models [33], namely that men will invest more in mating effort when they are more numerous than women. In this way, they ignore the dynamics that emerge when sex ratios, sex-structured pay-offs to mating strategies, time spent in parental care and sex-biased mortality differentials coevolve. For example, Henrich *et al*. [65] argue that when men are in abundance, they will invest more in indiscriminate competition over mates and provide less parental care than when they are scarce. In an ancillary prediction, they suggest that levels of violence increase cross-culturally as sex ratios rise, although this claim finds little support in the published literature [34].

Economists, demographers and sociologists, less familiar with the PI models that have dominated evolutionary social science for so long, have found results consistent with those reported here. Indeed, the relative abundance of men and women influences various other aspects of behaviour [38,66,67]. For example, marriage and marital fertility rates are higher when men are abundant but when the sex ratio is low, marriage incidence declines, female-headed households increase and non-marital fertility rates increase as men pursue concurrent relationships [68]. Additional evidence comes from the ethnographic record where an association of polygyny with low human sex ratios was identified over 40 years ago [69]. These findings lend support to the theoretical model (model 2) explored here.

Third, a major methodological advance of our work over much of the earlier work on gender variability is its focus on within-population variation. While between-population comparisons are immensely valuable for flagging interesting lines of investigation [22], they are typically marred by confounding factors such as religion, levels of economic development, political ideologies, legally observed marriage rules and disease levels, all of which might affect sex-specific mating strategies. Our work, by contrast, places the evolutionary analysis of sex-role variability firmly in an ethnographic and cultural context and finds little support for *consistent* differences in allocations between mating and parental effort between men and women across communities within a single cultural group. We maintain that looking at individual variability, where selection on phenotypic flexibility in mating and parental behaviour most likely takes place, is more persuasive than between-population comparisons, given the danger of committing an ecological fallacy [70].

Furthermore, by conducting our study within a single ethnic group, we provide some control for additional unmeasured and potentially confounding factors. This is particularly important in that we know that ASR is likely to shape sex differences in mating effort in conjunction with other factors, such as levels of paternity certainty, sex differences in the extent of parental care, and variation in quality across males and females that might reliably indicate future mating success.

Fourth, it is important to recognize that responses to the ASR may occur over evolutionary or ecological time scales (reviewed in [71]). The ASR model that we test here derives from a model [2] in which sex ratios influence the reproductive strategies of males and females as a result of feedbacks over evolutionary time, subject ultimately to Fisherian equilibrium. However, our study focuses on plasticity over an ecological time scale, and more specifically among communities that vary in ASR as a result of differential outmigration over a generation or less. Given the pronounced variability in human reproductive strategies, we maintain that evolutionary predictions are valid on a shorter time scale if the behaviours of interest are sufficiently facultative and the independent variables sufficiently varied, although for the most part the accuracy of predictions made over different time scales has been given insufficient attention [72]. We both demonstrate plasticity in human sex roles and suggest that this plasticity is adaptive with respect to community ASRs.

Finally, we turn to outstanding questions. First, we do not know which cues in the environment individual Makushi are responding to—do they gauge community sex ratios directly or are there other more salient social cues [73–75]? Second, why do women show no effect? Do women here benefit less from increasing mating effort in response to ASR than men? Are women instead remaining restricted in their sociosexuality to signal paternity certainty as a strategy to receive more paternal care? Recent evidence from birds suggests that this is possible [49]. Or are women responding in other ways, such as varying the traits they use to select partners? Finally, does culturally transmitted information condition the appropriate responses of women more so than of men, such that women's options are more constrained? The answers to these questions, either in general or specifically for the Makushi, are still unknown, but continued attention to developments in evolutionary theory will help provide insights into the environmental and/or culturally transmitted cues with which this variation is associated.

## 5. Conclusion

In summary, we show first that when men outnumber women (male-biased ASR), men's and women's willingness to engage in uncommitted sex are indistinguishable, a result challenging some persistent views on sexual behaviour. We also find that when men are in the minority (female-biased ASR), they are more willing to engage in uncommitted sex (less restricted sexual behaviour) than when they are numerous. As men become more abundant, they appear to reduce their mating effort and modify their behaviour to the desires of the limiting sex in response to the mating market and their place within it. This finding highlights an important point often overlooked in studies of sex differences. Because women pay higher reproductive costs (through gestation and lactation), it is argued that they are less likely to desert a mate or be interested in concurrent relationships. What is unrecognized is that males may also face steep reproductive costs that can constrain their willingness to pursue additional mates. When the pool of males is large, finding a female partner can be difficult such that existing partners become a valued resource. Thus, the frequency-dependent nature of optimal behaviour can, as shown here, have a major impact on sex-differentiated reproductive strategies.

## 6. Material and methods

### 6.1 Population

The Makushi inhabit the Rupununi savannahs of southwestern Guyana, Region 9 (figure 1). Living along the border with Brazil, this ethnic group shares cultural traits with other groups from the Xingu basin. They include shifting cultivation, a focus on bitter cassava, matrilocal marriage, the performance of bride service before marriage and fairly egalitarian gender relationships [76]. Whereas premarital sex is not disapproved of and is an expected avenue to secure a partner [51], the Makushi generally marry monogamously and extended families typically share one residential area [77]. Makushi marriages are endogamous, with mates are usually selected from within the village community [51]. As elsewhere in Guyana, differential migration affects family dynamics and has led to considerable between-community variation in ASR, as men and women search for economic opportunities.

Such migration strongly affects the availability of potential mates for endogamous marriage but men are still expected to perform bride service in order to marry. This traditionally involved a year of service by the prospective husband in which he cleared and farmed fields for his in-laws while building a new dwelling nearby for himself and his wife. Men and women typically marry only once or twice, and conventions are similar across all marriages, with men providing bride service and thereafter bearing considerable responsibility to provide for their wives and children (including stepchildren who are valuable helpers) through farming, fishing and various forms of wage labour [51]. At divorce, children remain largely the responsibility of the mother and her family, although they are provided for by a stepfather if she remarries.

### 6.2 Assessment of mating effort

Although sexual mores in most populations within Guyana, including Amerindian communities, are generally relaxed, direct quantitative measures of mating effort are potentially difficult to obtain. Accordingly, we used the (SOI) (see the electronic supplementary material for additional information, the list of questions and score calculation). The SOI is a seven-question instrument with proved reliability and validity [52,53]. It assesses sexual preferences and behaviour. In previous work (e.g. [53,57,58]), the SOI has been shown to be an indicator of indiscriminate mating, low parental care and high mating effort. Individuals who score high on this instrument are more willing to engage in uncommitted sex (unrestricted sociosexuality) and those who score low are less willing (restricted sociosexuality; [53]). Previous studies have confirmed that the SOI is a predictor of individual mating strategies—with those scoring high being more interested in short-term relationships (a mating-effort intensive strategy) and those scoring low more interested in long-term relationships [54–56].

We used ethnographic and demographic methods to conduct a full census of the first village in order to determine the ASR (table 1). We then administered the SOI to a randomly sampled minimum of 30 individuals (15 men and 15 women), aged between 18 and 45 years, from the village (table 1). This process was then continued across the subsequent seven villages, for a total of 8. Questions of a sensitive nature are found within the SOI and this has the potential to generate a response bias. In order to counter such bias, we took three steps: (i) a long (16 month) period of fieldwork during which community rapport could be built within each of the villages, (ii) gender-matched interviewers and interviewees, and (iii) the use of a non-verbal response card method [78] to ensure the privacy of the interviewee's response, even from the interviewer.

### 6.3 Statistical approach

We fit Gaussian models after applying a log transformation to the response SOI score and predictor ASR. The log transformation is variance-stabilizing for SOI scores, and the quantile–quantile plot for logSOI is consistent with a Gaussian distribution. A corresponding log transformation for the predictor ASR produced compatible scalings in the two-dimensional scatter plot (figure 2*b*). Because the dataset is structured with individuals nested within communities that differ in ASR, we use multilevel modelling [79]. While in some cases, it is appropriate to explore large set of models, following the advice of Burnham & Anderson [80], we keep our model set small and based on theory. Our model-fitting approach employs the AIC [81] to summarize the support for a given hypothesis by evaluating the relative evidence in favour of a corresponding model fitted to the data (model 1, model 2 or some combination; see [82] for a discussion on how to draw inferences from data in evaluating hypotheses). The AIC measures a goodness-of-fit versus complexity trade-off for each model, with lower values of AIC indicating better models. A model with an AIC of 10 or greater than that of the best model under consideration has essentially no support and may be omitted from further consideration because it fails to explain substantial variation in the data [80].

## Supplementary Material

I. Summary Details on the SOI and its Administration II. Sociosexual Orientation Inventory (Simpson and Gangestad 1991)

## Data accessibility

Data available from the Dryad Digital Repository: doi:10.5061/dryad.587v1.
