# Supplementary material for: Sex ratio effects on reproductive strategies in humans
Source: R Soc Open Sci. 2015 Jan 14;2(1):140402. doi: 10.1098/rsos.140402 (PMC4448795; doi:10.1098/rsos.140402)
Supplement: I. Summary Details on the SOI and its Administration II. Sociosexual Orientation Inventory (Simpson and Gangestad 1991) [file rsos140402supp1.docx]

**SUPPLEMENTAL MATERIAL TO SCHACHT AND BORGERHOFF MULDER**

Here we provide summary and additional information regarding the SOI (I) followed by the index itself (II).

**I. Summary Details on the SOI and its Administration**

At each village (n=8) we first conducted a full census to determine the ASR. We then randomly sampled a minimum of 30 individuals (n=300) from each village. Each interviewee was administered the SOI, which is a 7-question instrument with proven reliability and validity (Simpson and Gangestad 1989; 1991) in assessing sexual preferences and behavior. These ‘sociosexuality' scores are used to evaluate an individual’s willingness to engage in uncommitted sexuality activity. Individuals who score high on the SOI scale (termed “less restricted”) evince a reproductive strategy of multiple short-term relationships; those scoring low on the SOI scale (“more restricted”) prefer longer-term relationships. Because sensitive questions have the potential to generate a response bias we took three necessary measures: a) a long (16 month) period of fieldwork during which community rapport could be built across each of the villages, b) gender matched interviewers and interviewees, and c) the use of a nonverbal response card method (Lindstrom et al. 2009) to ensure the privacy of the interviewee’s response, even from the interviewer. A revised and expanded version of the SOI (SOI-R) was developed by Penke and Aspendorf (2008) to address questions of internal consistency and the mixture of response options. We decided not to use the SOI-R, in part because of the inappropriateness of several of the new questions for the Makushi context, and in part to retain comparability with other studies.

**II.** **Sociosexual Orientation Inventory (Simpson and Gangestad 1991)**

1. With how many different partners have you had sex (sexual intercourse) within the past year?

2. How many different partners do you foresee yourself having sex with during the next five years? (Please give a specific, realistic estimate)

3. With how many different partners have you had sex on one and only one occasion?

4. How often do you fantasize about having sex with someone other than your current dating partner? (Circle one).

1. never

2. once every two or three months

3. once a month

4. once every two weeks

5. once a week

6. a few times each week

7. nearly every day

8. at least once a day

5. Sex without love is OK.

1 2 3 4 5 6 7 8 9

I strongly disagree I strongly agree

6. I can imagine myself being comfortable and enjoying "casual" sex with different partners.

1 2 3 4 5 6 7 8 9

I strongly disagree I strongly agree

7. I would have to be closely attached to someone (both emotionally and psychologically) before I could feel comfortable and fully enjoy having sex with him or her.

1 2 3 4 5 6 7 8 9

I strongly disagree I strongly agree

**Scoring:** Item 7 should be reverse keyed prior to aggregating Items 5-7. The following weighting scheme should be used when aggregating components of the Sociosexual Orientation Inventory: SOI = 5 x (Item I) +1 X (Item 2) + 5 X (Item 3) + 4 x (Item 4) + 2 X (aggregate of Items 5-7). To ensure that Item 2 does not have disproportionate influence when constructing the composite, the maximum value of Item 2 should be limited to 30 partners foreseen in college samples.
